# Genetic Association Analysis of Copy Number Variations for Meat Quality in Beef Cattle

**DOI:** 10.3390/foods12213986

**Published:** 2023-10-31

**Authors:** Jiayuan Wu, Tianyi Wu, Xueyuan Xie, Qunhao Niu, Zhida Zhao, Bo Zhu, Yan Chen, Lupei Zhang, Xue Gao, Xiaoyan Niu, Huijiang Gao, Junya Li, Lingyang Xu

**Affiliations:** 1 State Key Laboratory of Animal Biotech Breeding, Institute of Animal Sciences, Chinese Academy of Agricultural Sciences, Beijing 100193, China; wujy0610@163.com (J.W.); zhubo525@126.com (B.Z.); zhanglupei@caas.cn (L.Z.); lijunya@caas.cn (J.L.); 2 College of Animal Science and Veterinary Medicine, Shanxi Agricultural University, Jinzhong 030801, China

**Keywords:** copy number variation, association analysis, meat quality, beef cattle

## Abstract

Meat quality is an economically important trait for global food production. Copy number variations (CNVs) have been previously implicated in elucidating the genetic basis of complex traits. In this article, we detected a total of 112,198 CNVs and 10,102 CNV regions (CNVRs) based on the Bovine HD SNP array. Next, we performed a CNV-based genome-wide association analysis (GWAS) of six meat quality traits and identified 12 significant CNV segments corresponding to eight candidate genes, including *PCDH15*, *CSMD3*, etc. Using region-based association analysis, we further identified six CNV segments relevant to meat quality in beef cattle. Among these, *TRIM77* and *TRIM64* within CNVR4 on BTA29 were detected as candidate genes for backfat thickness (BFT). Notably, we identified a 34 kb duplication for meat color (MC) which was supported by read-depth signals, and this duplication was embedded within the keratin gene family including *KRT4*, *KRT78*, and *KRT79*. Our findings will help to dissect the genetic architecture of meat quality traits from the aspects of CNVs, and subsequently improve the selection process in breeding programs.

## 1. Introduction

Growing consumer demand for high-quality beef has promoted the selection of cattle for meat quality traits [1,2]. Backfat thickness (BFT) and marbling score (MS) represent the fat content and influence meat palatability. Additionally, pH, meat color (MC), and fat color (FC), also indicate beef quality by affecting visual appeal, consumer acceptance, and freshness [3,4,5,6,7,8]. Over past decades, quantitative trait loci (QTLs) mapping has identified numerous genomic regions associated with economically important traits in cattle. However, progress characterizing the genetic basis of meat quality (exhibiting low to moderate heritability) has been hampered by challenges in phenotype measurement. Several studies have identified lists of candidate genes for meat quality in various cattle populations [6,9,10,11]. For instance, *GABRB*, *PPP3R1* and *CRYAB*, etc. have been identified as candidate genes related to MC and REA [12,13,14]. Meanwhile, the application of high-throughput approaches has proven useful for understanding the genetic basis [15,16,17,18] and genetic improvement of meat quality in cattle [19]. 

In recent years, genome-wide association studies (GWAS) have pinpointed thousands of variants of human diseases [20,21,22]. However, the elucidation of the genetic architecture of complex traits has mainly relied on single nucleotide polymorphisms (SNPs), while few investigations of copy number variations (CNVs) have been explored [23,24,25]. CNVs, including deletions and duplications, represent a vital source of genetic diversity which can alter gene structure and expression, potentially with stronger effects than SNPs [26,27]. CNVs have been linked to complex traits such as hair color [28], obesity [29], spina bifida [30], vesicoureteral reflux [31], and seizure disorders [32] in humans. While studies of CNVs in livestock have received much attention, relatively few studies have aimed to detect candidate CNVs for economically important traits via association analysis on a genome-wide level. To date, CNVs have shown potential relevance to traits like BFT and REA [33,34], fat deposition [35], and coat color [36,37] in pigs, sheep, goats, and dogs. Existing studies have mainly focused on the relationship between CNVs and growth traits in cattle [38,39,40,41,42].

So far, many previous studies have reported on causal genes for meat quality via SNP makers in cattle. For instance, one study has utilized SNPs to map the QTL on BTA6 in approximately 3500 cattle from multiple cattle breeds, encompassing *NCAPG* and *LCORL* related to BFT [43]. In particular, the role of the *ACDSB* gene in the β-oxidation process, which in turn affects meat color stability, has already been reported with single and multiple-trait SNP-GWAS in Nellore cattle [12]. Also, a recent study of Hanwoo beef cattle described *ENPP2*, *POLI*, and *CPAMD8* as candidate genes for MC and FC traits [44]. A separate study identified several key genomic regions accounting for over 1% of the total genetic variance of traits like BFT and MS using GWAS of Montana Tropical Composite beef cattle [45]. Moreover, a network analysis revealed numerous candidate genes, including *CAPN1*, *CAST*, *MYOM1*, and *CALCOCO1*, involved in metabolic and cellular processes that have potential impacts on the meat quality of Angus cattle [46].

However, few studies have conducted genome-wide CNV analyses for assessing meat tenderness in Nelore cattle [47]. Therefore, a comprehensive genome-wide study of CNVs for more meat quality traits in other cattle populations remains to be explored. The objective of this study was to characterize the CNVs using a high-density SNP array. By utilizing CNV-based GWAS and region-based CNV association analysis, we identified several candidate CNV segments for meat quality with multiple strategies. The identified candidate genes with copy number changes may offer potential genetic markers for the selection of meat quality traits in cattle.

## 2. Material and Methods

### 2.1. Animal Populations

The studied population consisted of 1466 Chinese Simmental beef cattle, which were born between 2008 and 2022 in Ulgai, Inner Mongolia. All cattle were uniformly managed and fed a standardized diet based on the total mixed ratio (TMR) recommended at an average age of 22 months, and then they were transferred to Inner Mongolia ZhongAo Food Co., Ltd. (Chifeng City, Inner Mongolia Autonomous Region, China) for slaughter via a consistent and streamlined process. No ethics statement was required for the collection of the genetic material. The data from animals included in this study were derived from previous analyses that obtained specific permissions.

Blood samples were obtained with routine quarantine inspection before slaughter. Samples were genotyped using an Illumina BovineHD SNP array containing 777,962 SNPs. The SNP positions were obtained according to the ARS-UCD1.2 genome assembly. More details were described in our previous study [48].

### 2.2. Phenotype Measurement

Six meat quality traits, namely fat color (FC), meat color (MC), marbling score (MS), backfat thickness (BFT), pH, and ribeye area (REA), were selected for the subsequent analyses. The REA of the cattle was measured at the end of the fattening period between the 12th and the 13th rib (longissimus thoracis muscle area). Afterwards, the carcass was chilled for 48 h (partly 24 h post-slaughter). BFT was measured after slaughter in the same position as the REA. The pH was measured at three locations of steak from the twelfth rib at slaughter using a Mettler Toledo pH meter (Mettler Toledo, Greifensee, Switzerland). Marbling score was assessed from 1 to 5. Meat color was graded in seven categories against a standard color palette, from pale (grade 1) to dull red (grade 7). Fat color was graded in seven categories against a standard color palette. More details of the measurement of phenotypes for meat quality were presented in previous analyses [24,25].

The significant effects (*p* < 0.05) were tested using variance analyses. A general linear model was used to analyze the traits so that batch and sex were considered as the fixed effects, and weight before fattening and PCs 1–3 were regarded as the covariates. The fixed effects and covariates were adjusted in the linear regression model and the residuals were further considered as adjusted phenotypes for the association test. Heritability was calculated using GCTA [49].

### 2.3. Quality Control and Detection of CNVs 

DNA was extracted from blood and genotyped using the Illumina Bovine HD 770K Bead chip (Illumina Inc., San Diego, CA, USA). Genome Studio 2.0 were employed to extract signal intensity data including Log R Ratio (LRR), B allele frequency (BAF), and population frequency of the B allele (PFB) file. CNVs were detected using PennCNV v.1.0.5, which is based on a hidden Markov model (HMM) [50]. The PFB file (containing the position marker) was obtained according to a previous study [40], and the genomes’ positions were converted from UMD3.1 to ARS-UCD1.2 using a liftover tool. The GC file (generated by calculating the GC content of the 1 Mb genomic regions surrounding each marker) was obtained from UCSC genome bioinformatics. The final CNVs were obtained after quality control based on cutoff values (LRR standard deviation 0.30, BAF drift 0.01, and waviness factor 0.05) using the filter_cnv.pl function.

### 2.4. CNV Compilation

The CNVs were merged into CNV regions (CNVRs) using HandyCNV [51]. The “call_cnvr” option identified CNVRs through the union of CNV sets that overlapped by at least one base pair. CNVs of overlapping gain and were combined into a single region to account for genomic regions where both events had happened (“mixed” CNVR).

### 2.5. CNV-Based GWAS

The CNV-based GWAS were performed using a CNVRanger package [52] between each trait and CNV state based on a general linear model [53]. For association analyses, CNVRanger re-established the CNV segments with the probes, while the probe with the lowest *p*-value was considered as an indicator for this segment. The significance level of the FDR correction (FDR  <  0.05) was used to determine the associated CNV segments.

### 2.6. Region-Based GWAS

We then employed CNVtools, a tool that amalgamates signals from multiple CNV probes to generate a one-dimensional LRR profile fitted with Gaussian mixtures [54]. This method produces a regional estimate of the cumulative CNV burden. The Bayesian information criterion (BIC) was utilized to select the optimal number of mixture components. Copy number genotypes were assigned to each locus of the individual cattle. Standard regression was used to test genetic associations between CNV burden estimates and meat quality traits. We further applied a likelihood ratio test to principal components of the adjusted phenotypes, identified via linear discriminant analysis. Significant association regions were defined based on a threshold of *p* < 0.05.

### 2.7. CNV Validation Using Whole-Genome Sequencing

To confirm the identified CNVs, we further carried out validation using a ~20× whole-genome sequencing (WGS) dataset [41]. To measure consistency, the overlapping regions were estimated and visualized using an Integrative Genomics Viewer (IGV) [55]. Then, we randomly selected CNV carriers from cattle with the candidate CNVs and normal cattle from the sequencing datasets and observed the distribution of the readings. Additionally, the reference genome was segmented into non-overlapping sliding windows according to the target CNV fragment size. The raw read-depth was then calculated for each window using bedtools (v2.17.0) to assess the number of mapped reads within the boundaries of each window [56]. Also, we compared the results of the current study with high-quality CNVR datasets estimated from WGS (more than 12× depth) of 171 distinct populations, including both Bos taurus and Bos indicus [57].

### 2.8. Annotation of CNVRs and Functional Enrichment Analyses

Next, we explored the candidate genes based on the identified CNVs via a UCSC genome browser (ARS-UCD 1.2). Genes overlapping with the detected significant CNV segments were extracted for further analysis using bedtools [56]. GALLO software was used to annotate the significant CNV regions identified by GWAS and their upstream/downstream 250 kb intervals based on the cattle QTLdb [58,59]. Additionally, QTL enrichment analysis based on a hypergeometric approach was performed to directly connect the QTL regions identified in CNV-based GWAS overlapping with QTLs, based on the cattle QTLdb. A gene set enrichment analysis of the candidate genes was also conducted using g:Profiler [60]. The significance of GO terms was determined by the adjusted threshold (*p* < 0.05). STRING (http://string-db.org/, accessed on 8 September 2023) was utilized to search for enriched pathways and protein domains, and identify PPI networks based on candidate genes.

## 3. Results

### 3.1. The Detection of CNVs and CNVRs

**Figure 1:**
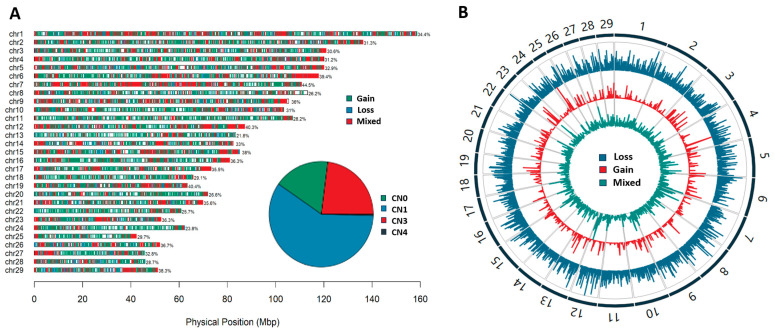
(**A**) Landscapes of CNVR distribution in 29 autosomes and pie charts for the proportions of four types of CNVs with different copy numbers. (**B**) Manhattan plots showing the −log10 *p*-values of the frequency of CNVRs.

**Figure 2:**
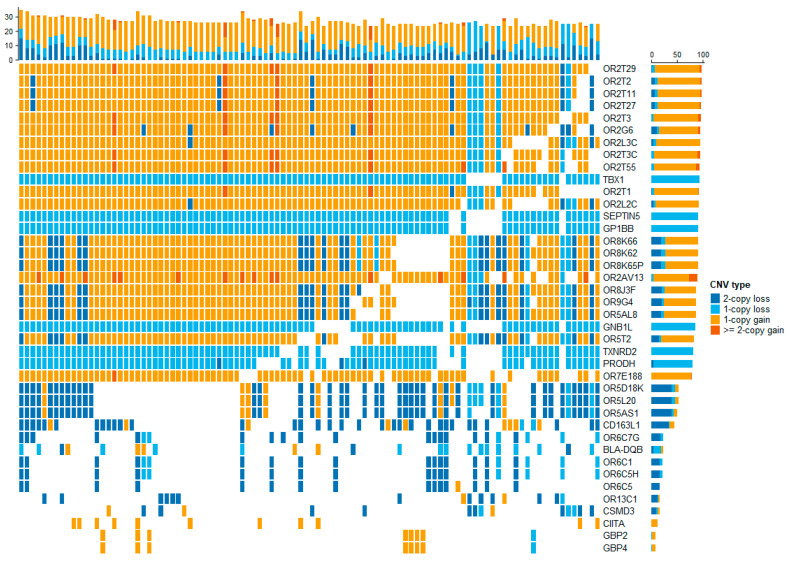
Stacked bar plots showing the genes with highest frequency overlapping with CNVRs; the top and right section of the plots show the number of altered genes per sample and the number of altered samples per gene, respectively.

We detected 217,369 CNVs across 29 autosomes in 1466 individuals. After quality control, a total of 112,198 high-confidence CNVs in 1361 individuals was retained for subsequent analyses. We found that deletions (76.6%) were more abundant than duplications (23.4%) across the genomes. Respectively, the length of the duplications was between 634 bp and 1.6 Mb (3–304 probes), while deletions ranged from 602 bp to 1.7 Mb (3–509 probes). The fraction of homozygous CNVs (CN 0 or 4) was low (17.3% or 0.4%; Figure 1A). Overall, these CNVs were merged into nonredundant CNVRs. A total of 10,102 CNVRs were identified including 5934 losses, 2315 gains, and 1853 mixed CNVRs, which were uniformly distributed across autosomes (Figure 1A). We found that the highest percentage of CNVR length (44.5%) was located on BTA7. Moreover, we observed the highest frequencies of loss CNVRs on BTA1 and BTA12. For gain/mixed CNVRs, the highest frequencies were observed on BTA4, BTA25, BTA12 and BTA23 (Figure 1B). Based on the identified CNVRs, we searched the genes using a UCSC genome browser (ARS-UCD1.2). We obtained 4207 CNVRs overlapping with 7074 genes, while 5895 CNVRs were located in intergenic regions. Notably, we found the CNVRs with high frequency overlapping with highly variable genes such as the OR gene family, *TBX1* and *CSMD3* (Figure 2).

### 3.2. CNV-Based Association Analyses for Meat Quality

To investigate the impact of CNVs on meat quality, we conducted a genome-wide association analysis using CNV segments. The estimated heritability was consistent with previous studies reporting that most traits have low heritability, except fat color (FC), which has moderate heritability (0.40) [6,10,11]. Descriptive statistics and heritability estimates of meat quality traits are presented in Table S1.

**Figure 3:**
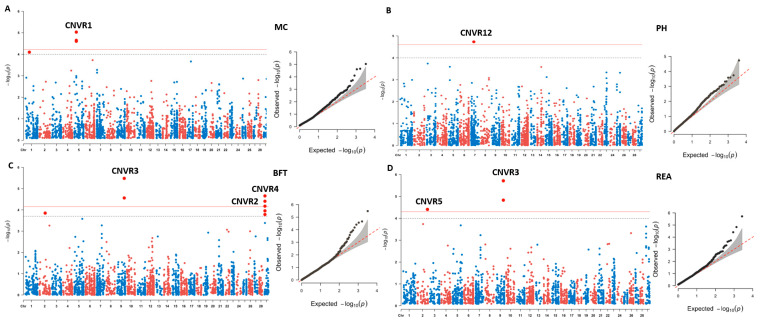
Manhattan and QQ plots showing the −log_10_ *p*-values for CNV-based GWAS. (**A**–**D**) Genome-wide association results of CNV segments for MC, pH, BFT and REA, respectively. The red and black lines represent the genome-wide significance threshold at *p* value = 4 × 10^−05^, 2 × 10^−04^.

We identified six CNV segments located on BTA1, BTA2, BTA5, BTA6, BTA9, and BTA29 using CNV-based GWAS. In this study, the significant CNV segments with adjacent probes were merged together, from which we identified CNVR1 and CNVR12, uniquely associated with MC and pH, respectively (Figure 3A,B). CNVR3, located at 92.8 Mb on BTA9, was significantly associated with both BFT and REA (Figure 3C,D). Furthermore, six of the candidate CNV segments overlapped with five genes, including *KRT4*, *KRT78*, *KRT79*, *TRIM77*, and *TRIM64* (Table 1). Additionally, we identified six CNV segments relating to MS and FC using a suggested threshold (FDR cutoff of 0.1, −log_10_P = 3.69; Figure S1A,B). Particularly, we found three significant signals in CNVR7, CNVR10, and CNVR11 related to MS and FC, which embodied *PCDH15*, *CSMD3*, and *OR56B2C*, respectively.

### 3.3. Functional Annotation

To examine the functional annotation, we further intersected associated CNV segments with substantial QTLs based on the cattle QTLdb. Strong correlations were observed for milk tetracosanoic acid content- and milk fat percentage-related traits (Figure S2A,B). Furthermore, we performed Gene Ontology (GO) and Reactome (REAC) pathway enrichment analyses of the candidate genes. We identified biologically relevant clusters including potential factors for regulating meat skin coloration, such as keratin filaments and intermediate filaments (Figure S3A), and pathways involved in keratinization and developmental biology in the REAC database. The GO terms and significant pathways are listed in Table S2. Additionally, we utilized the STRING database to investigate pathways and protein domains based on the genes annotated within a 1 Mb window of significant CNV segments from the CNV-based GWAS. Interestingly, we observed three identified networks associated with intermediate filaments and keratin (Figure S3B, Table S3).

### 3.4. Region-Based Association Analyses of Candidate CNV Segments

**Figure 4:**
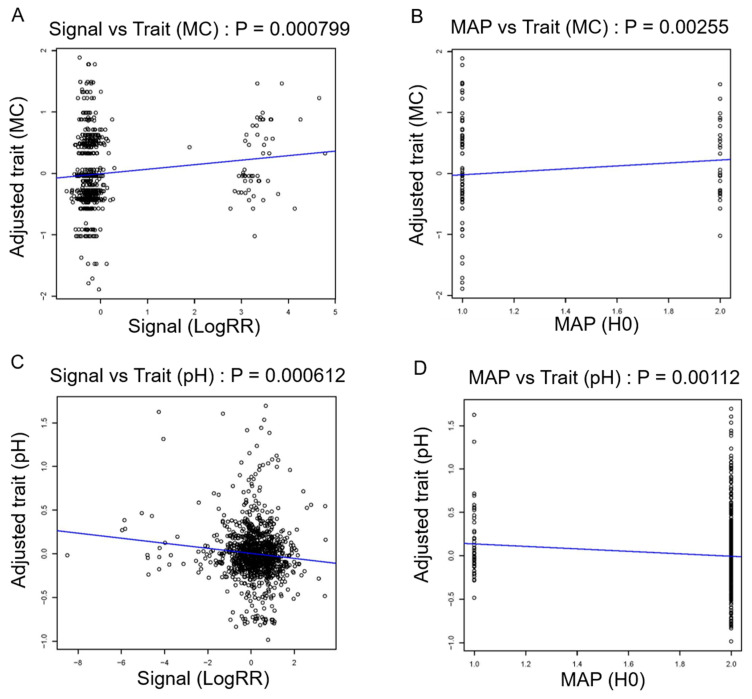
Region association tests of CNV segments at 27 Mb on BTA5 and 4 Mb on BTA7 for MC and pH. (**A**) The adjusted traits against signals (LogRR) for CNV segments at 27 Mb on BTA5. (**B**) The adjusted traits against copy number states (MAPs) estimated for CNV segments on BTA5 using mixture model assignment. (**C**) The adjusted traits against signals (LogRR) for CNV segments at 4 Mb on BTA7. (**D**) The adjusted traits against copy number states (MAP) estimated for CNV segments on BTA7 using mixture model assignment.

To further investigate the significant association of CNV segments, we explored potential correlations between raw Log R Ratio (LRR) values and corresponding meat quality traits with a robust statistical analysis framework. After a likelihood ratio test, we found significant associations for MC, MS, pH, and BFT based on the nominal *p* values. As expected, we confirmed a duplication CNVR1 with 34 kb on BTA5 (Figure 4A,B) and CNVR12 with 7 kb (Figure 4C,D) for MC and pH, while other peak signals were observed within CNVR4 (Figure S4A,B) and CNVR8 (Figure S4C,D). In contrast, we observed CNVR5 for REA, which was detected using CNV-based GWAS (*p* = 6.22 × 10^−05^), though it did not show significant association signals using region-based association analysis (*p* = 0.0629).

### 3.5. Analysis of Candidate CNVs Using Whole-Genome Sequencing Data

**Figure 5:**
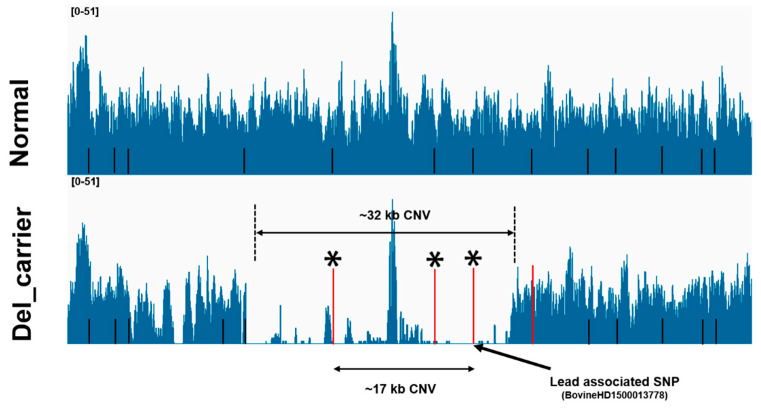
Identification of a 32 kb deletion (BTA 15:47,429,054-47,461,008) with WGS using IGV. The 17 kb deletion (BTA 15:47,438,725-47,455,722) was detected with CNV-based GWAS, and the main associated SNP was BovineHD1500013778. Four SNP probes (marked in red) mapped by CNV-GWAS were located within the CNVR12, while three probes (marked with black asterisks) were supported by read-depth signals.

We then validated the candidate CNVs identified by high-density SNP array using whole-genome sequencing (WGS) data from the same cattle. As shown by the Integrative Genomics Viewer (IGV), the accuracy levels of CNV calling obtained from the array were consistent with the whole-genome sequencing data [41]. Among twelve significant CNV segments based on CNV-based GWAS, five CNV segments were confirmed by read-depth signals from sequencing cattle. In this study, we identified that the OR56B2C gene in CNVR11 (17 kb) was potentially related to FC, as read-depth signals also confirmed the occurrence of a 32 kb CNV (Figure 5). Moreover, we found strong read-depth signals around the region of CNVR8 for MS, which is located in exon regions of *PCDH15* gene (Figure S5). Additionally, the distribution of the normalized read-depth of the CNV segment suggested the presence of CNVR3, which was associated with BFT and REA (Figure S6). In addition, we compared our results with the high-quality CNVRs detected with WGS from different populations in a previous report [57]. Of those, CNVR1, CNVR2, CNVR9, and CNVR12 were shared by both Bos taurus and Bos indicus (Table S4). Notably, we especially found CNVR2 and CNVR4 for BFT overlapping with several CNVRs detected with WGS, which includes *TRIM77* and *TRIM64*.

### 3.6. 34 kb-Duplication as a Candidate CNV Segment Related to Meat Color

**Figure 6:**
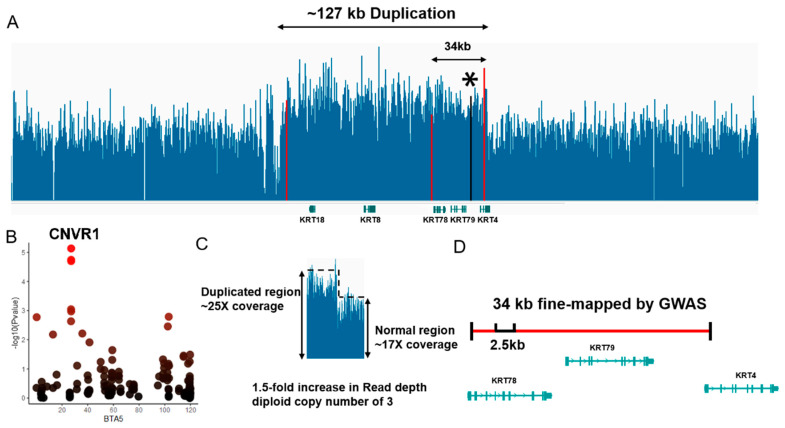
(**A**) Characterization of read-depth signals with duplication at the sequencing depth using IGV, and the regions encompassing this duplication, represented by read-depth coverage. The boundaries of CNV (127 kb) and CNVR1 (34 kb) detected with CNV-based GWAS are marked in red. The main associated SNP probe for 34 kb duplication is marked with a black asterisk. (**B**) Region plots showing CNVR1 for MC (on BTA5) identified using CNV-based GWAS. From red to black the color of different dots corresponding to the level of significance of CNVRs. (**C**) The difference in sequencing depth signals between the CNV region and the normal region. (**D**) Schematic diagram of a zoomed-in 34 kb duplication (BTA 5:27085360-27119759) showing 2.5 kb CNV overlapping with CNVRs from WGS samples of multiple cattle populations.

We confirmed a ~127 kb duplication encompassing the keratin gene family *KRT4*, *KRT8*, *KRT18*, etc. (Figure 6A). Remarkably, the main associated probe (BovineHD0500007947, −log_10_P = 5.02) was located within CNVR1, which was validated with read-depth signals from sequencing data. Using CNV-based GWAS and region-based association, we further identified an association signal at 27.1 Mb on BTA5 for MC, which was overlapped with *KRT78*, *KRT79* (Figure 6B). The distribution of normalized read-depth signals for the 34 kb region from duplication-carriers indicates this multi-allelic locus has copy number (CN) changes (Figure 6C). In addition, we observed ~2.5 kb regions with CNVs at BTA 5:27,087,501-27,090,000 in both Bos taurus and Bos indicus (Figure 6D, Table S4). Our results confirm the presence of 34 kb duplication based on whole-genome sequencing analysis and suggest that *KRT78*, *KRT79*, and *KRT4* with copy number changes are promising genes for affecting MC. 

## 4. Discussion

Our study performed high-resolution CNV mapping for 1466 beef cattle using a high-density SNP array. For the CNVRs identified with high frequency, we obtained several candidate genes, including olfactory receptor gene family *OR2T29* and *OR2T2*, as well as *TBX1* and *CSMD3*. By conducting a comprehensive CNV-based GWAS, we identified a total of twelve candidate CNV segments related to meat quality traits. Additionally, we compared the results of an SNP-based GWAS with the CNVs identified in the current study using the same cattle population; we actually, for the first time, revealed several genes with copy number changes which have not been detected with SNP-based GWAS, suggesting that CNVs contribute to the potential lack of heritability of meat quality [25,61]. We believe our findings will contribute new insights into the genetic architecture of meat quality traits, as some CNVs may not be flagged by SNPs in cattle genomes [62].

It was noted that the detection of CNV segments for meat quality traits using genome-wide association analysis has been demonstrated in many previous studies [52,53,63,64]. In fact, CNVRanger divided potential CNV segments across genomes using a single probe and chose candidate CNVs based on the probes within segments that had the lowest *p*-value. Moreover, we implemented a robust likelihood ratio test (LRT) on raw LRR values for the candidate regions identified using CNV-based GWAS [54,65]. LRR is an approximation of the actual copy number and is affected by noise [66,67,68]. By incorporating this LRT and read-depth signals from sequencing analysis, our study enhances the accuracy of detecting candidate CNV segments CNV-based association analyses [52].

In the present study, we used WGS data from the same population of cattle based on read-depth signals to validate their CNVs, and compared them to the occurrence of CNVRs from WGS samples of multiple cattle populations. Strikingly, among these twelve CNVRs, four candidate CNV segments were found to be partially overlapped with the CNVRs of high confidence from other cattle populations. Eventually, we characterized CNVR1 and confirmed the presence of the ~12 kb CNV at 27.119 Mb on BTA5 in Bos taurus and Bos indicus based on read-depth signals which have undergone selection in a previous report [57]. The previous study suggests that among three KRT gene families overlapping with CNVR1 could have a role in the development of skin. For instance, mutations in the *KRT4* gene have been proven to encode mucosa-specific keratins which may cause white sponge naevus in humans [69]. Meanwhile, variants overlapping with the *KRT79* gene lead to the destabilization of K14 in sebocytes, attenuating sebocyte differentiation and lipid content in the skin [70]. In livestock, an overexpression of the *KRT4* gene was found to bind Tβ4, mediating the ERK signaling pathway in cashmere goats, which might control the developmental patterning of hair follicles [71]. Previous studies also found that the expression levels of *KRT4* and *KRT78* were higher in telogen compared to catagen and anagen through analysis of the transcriptome data of yak hair follicles at different developmental stages [72]. Despite the fact that many previous findings have shown that these three genes encoding keratin filament may be related to hair generation, the functional activity of KRT genes and the CNV dosage effect of KRT for MC still need to be fully examined by multi-omic strategies and molecular experiments. 

We also identified several CNV segments for BFT and MS, which were overlapping with genes such as *PCDH15*, *CSMD3*, *TRIM77*, and *TRIM64*. For MS, the *PCDH15* gene, which encodes protocadherin, has been reported to play an essential role in the stereocilia development of mice and glutamate levels [73,74,75]. Moreover, two recent studies detected that *PCDH15* genes were related to meat production in lambs and Tibetan sheep [76,77]. *CSMD3* was reported to have an impact on autistic spectrum disorders and immunity via QTL information [78,79]. Additionally, *TRIM77* and *TRIM64* are members of the TRIM family of E3 ubiquitin ligase proteins. Specifically, these genes play an important role in foam cell formation and cytokine production via the NF-κB signaling pathway. Pathway analysis of the candidate genes highlighted several processes for intermediate filament regulation. Interestingly, a recent study reported that an overexpression of *TRIM64* suppresses lipid accumulation [80]. While the biological functions of *TRIM64* have been partially explored regarding cancers, these genes in livestock remain unknown [80,81]. Our study revealed several CNVs corresponding to *TRIM77* and *TRIM64* for BFT, suggesting that these two genes may be involved in lipid metabolism. 

Notably, we found that this CNV segment harbored a 3.5 kb duplication (BTA 29:5,239,001–5,242,500), which have been indicated that under selection for climatic adaption in previous study [57]. Our strategy by integrating phenotypic with association for CNVs can help to clarify effects of new variants on complex traits. Our findings revealed several novel CNVs for meat quality traits in cattle. With advances in multi-omics and functional annotation [82,83], further studies are required to assess the impacts of CNVs on gene expression and their functional regulation, with experimental validations of the complex traits of farm animals.

## 5. Conclusions

Our study identified twelve candidate CNV segments for meat quality traits by using CNV-based GWAS of beef cattle. By integrating region-based association and sequence validation based on read-depth signals, we wholly obtained two candidate CNV segments (corresponding to KRT and TRIM) for MC and BFT. Moreover, we reported a 34 kb duplication on BTA5 harboring *KRT4*, *KRT78*, and *KRT79* for MC. Our findings provide novel insights into elucidating the genetic basis of meat quality traits from the aspects of CNV and may offer further potential markers for selection during the breeding process of farm animals.

## Figures and Tables

**Table 1:** Summary statistics of the CNV segments associated with meat quality traits and candidate genes.

Traits	CNV Segment	BTA	Location	Type	*p* Value	FDR	Candidate Gene
MC	CNVR1	5	27,085,360–27,119,759	Duplication	9.28 × 10^−06^	0.033	*KRT4*, *KRT78*, *KRT79*
BFT	CNVR2	29	5,171,306–5,281,082	Duplication	2.20 × 10^−05^	0.030	
	CNVR3	9	92,831,482–92,843,287	Duplication	3.30 × 10^−06^	0.006	
	CNVR4	29	5,287,673–5,295,854	Duplication	9.28 × 10^−06^	0.032	*TRIM77*, *TRIM64*
REA	CNVR3	9	92,831,482–92,843,287	Duplication	1.93 × 10^−06^	0.006	
	CNVR5	2	129,599,911–129,603,637	Deletion	6.22 × 10^−05^	0.042	
MS	CNVR6	9	30,539,303–30,579,950	Deletion	6.22 × 10^−05^	0.087	
	CNVR7	26	4,750,372–4,775,984	Deletion	1.23 × 10^−04^	0.099	*PCDH15*
	CNVR8	20	60,391,422–60,425,423	Both	1.15 × 10^−04^	0.099	
	CNVR9	1	37,762,712–37,775,698	Both	5.84 × 10^−05^	0.087	
FC	CNVR10	14	51,244,826–51,249,961	Deletion	5.58 × 10^−05^	0.077	*CSMD3*
	CNVR11	15	47,438,725–47,455,722	Both	3.10 × 10^−05^	0.077	*OR56B2C*
PH	CNVR12	7	40,149,305–40,156,641	Deletion	9.28 × 10^−06^	0.075	

## Data Availability

The datasets used and analyzed during the current study are available from the corresponding author on academic request.

## Supplementary Materials

The following supporting information can be downloaded at: https://www.mdpi.com/article/10.3390/foods12213986/s1, Figure S1. Manhattan plots showing the −log_10_ *p*-values for CNV-based GWAS; Figure S2. QTL enrichment results based on genes identified in CNV-based GWAS; Figure S3. Functional enrichment of candidate genes obtained through CNV analysis; Figure S4. Region association of BFT and MS for CNV segments at 5 Mb on BTA29 and 4 Mb on BTA26; Figure S5. Identification of the 34 kb duplication (BTA 20:60391422-60425423) based on WGS of a subject using the IGV program; Figure S6. Identification of an 11.8 kb duplication (BTA 9:92831482-92843287) based on WGS of a subject using the IGV program; Table S1. Descriptive statistics of six meat quality traits; Table S2. The GO and REAC enrichment analyses of gene mapped by CNV-based GWAS; Table S3. STRING enriched pathways and protein domains for CNV candidate genes related to MC; Table S4. Overlapped variants compared with CNVR datasets in cattle.

## Abbreviations

CNV: copy number variation; QTL: quantitative trait locus; GWAS: genome-wide association study; BTA: bos taurus autosome; CNVR: copy number variation region; IGV: integrative genomics viewer.
