# Supplementary material for: Genetic Association Analysis of Copy Number Variations for Meat Quality in Beef Cattle
Source: Foods. 2023 Oct 31;12(21):3986. doi: 10.3390/foods12213986 (PMC10647706; doi:10.3390/foods12213986)
Supplement: Supplementary file 1 [file foods-12-03986-s001.zip › foods-2643403-supplementary.pdf]

Supplementary Material

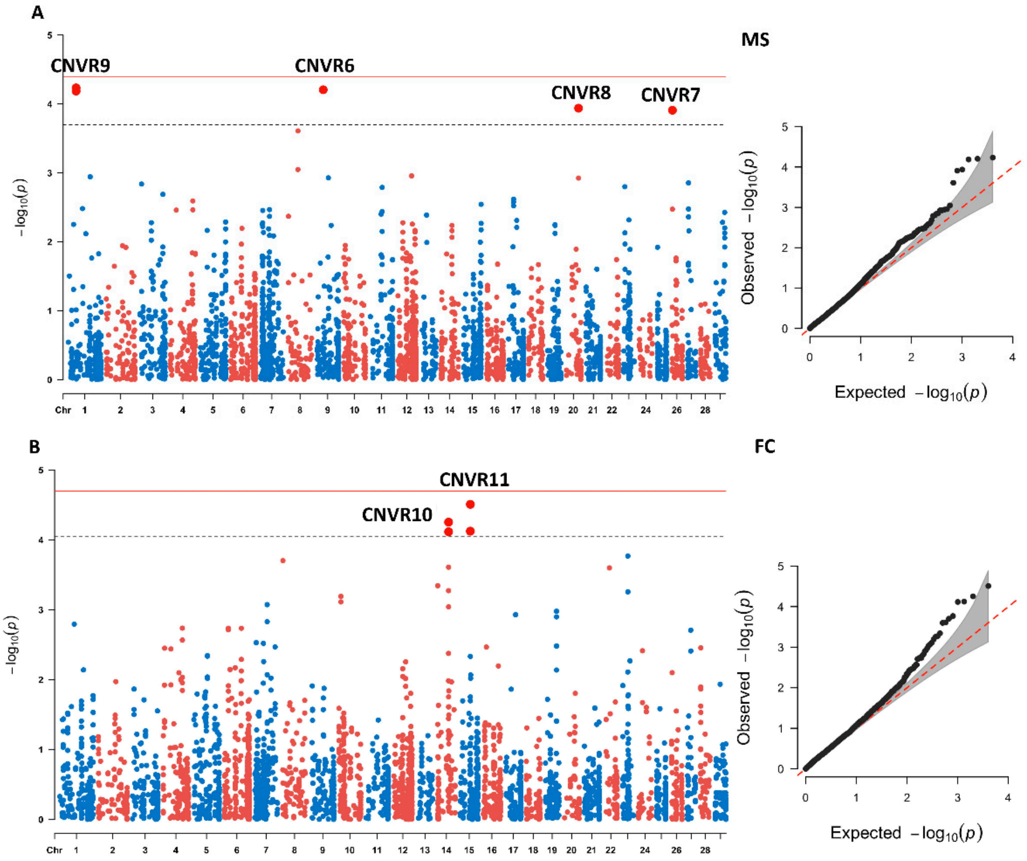

Figure S1. Manhattan plots showing the $-\log_{10}$ *p*-values for CNV-based GWAS. (**A**) Genome-wide association results for CNV segments for MS. (**B**) Genome-wide association results for CNV segments for FC. The red and black lines represent the genome-wide significance thresholds at *p* value = 4×10^{-05} and 2×10^{-04}, respectively.

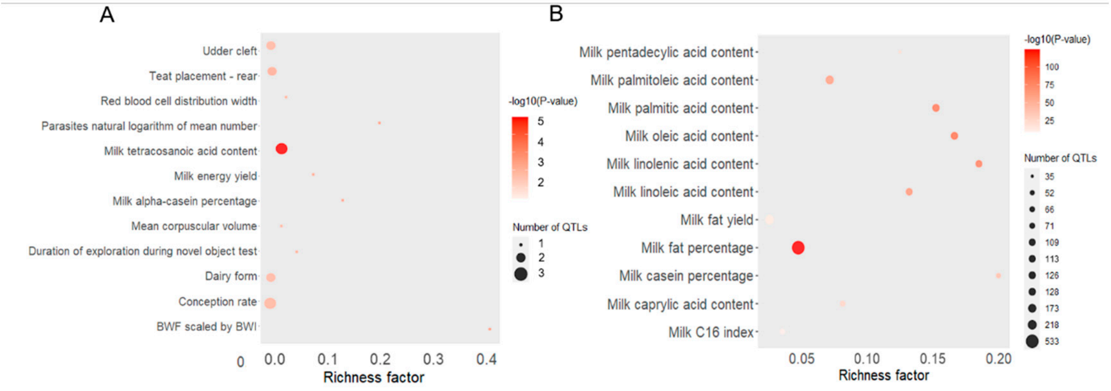

Figure S2. QTL enrichment results based on genes identified in CNV-based GWAS. (**A**) QTL enrichment analyses of significant CNVs for BFT. The richness factor was obtained using the ratio of the number of QTLs annotated in the candidate regions and the total number of each QTL. (**B**) QTL enrichment analyses of significant CNVs for MS.

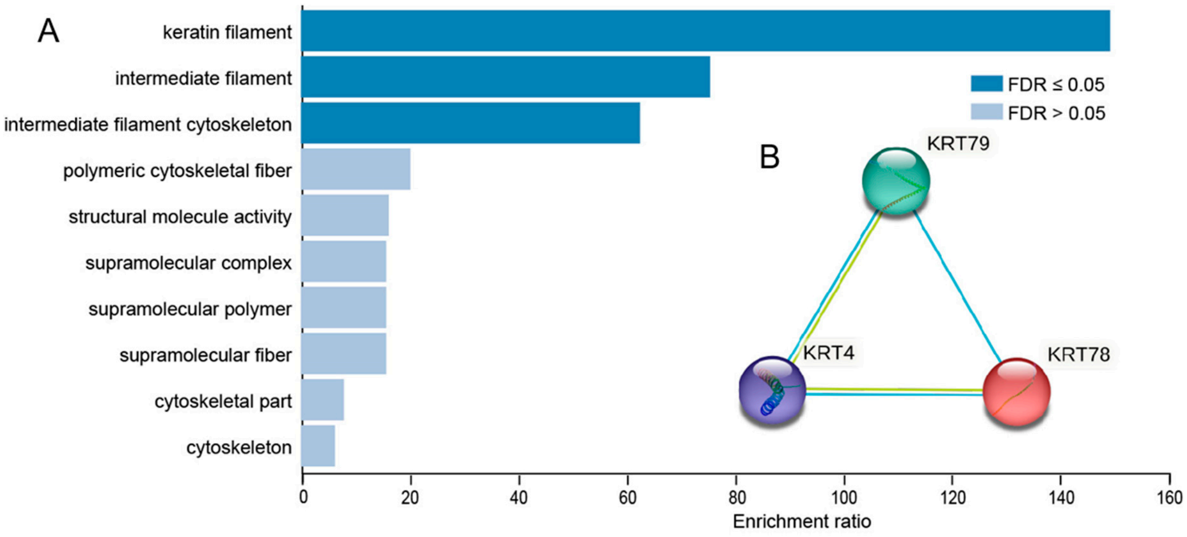

Figure S3. Functional enrichment of candidate genes obtained through CNV analysis. (**A**) Enrichment of GO terms associated with genes within CNVR1 (FDR < 0.05). (**B**) STRING enriched pathways and protein domains for CNVRs and candidate genes related to MC.

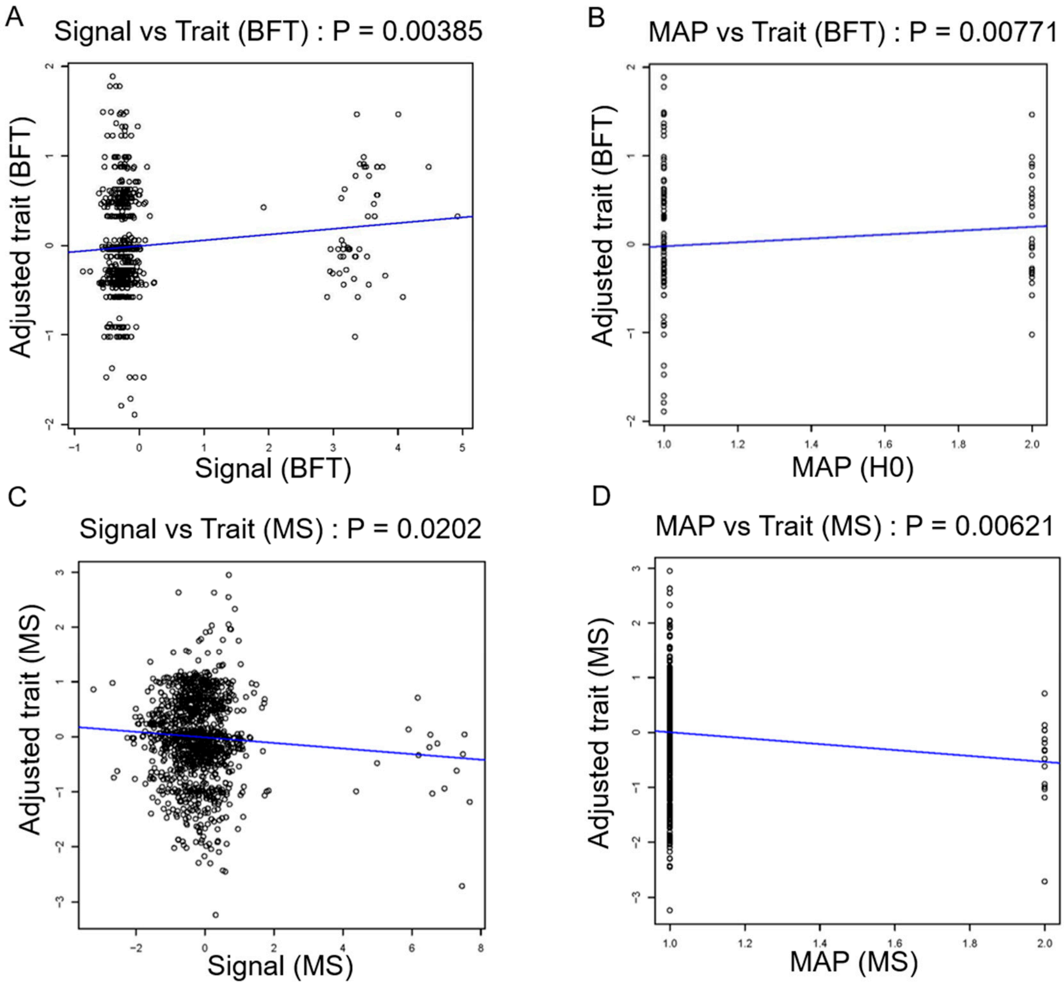

Figure S4. Region association of BFT and MS for CNV segments at 5 Mb on BTA29 and 4 Mb on BTA26. (**A**) The adjusted traits against signals (LogRR) for CNV segments related to BFT at 5 Mb on BTA29. (**B**) The adjusted traits against copy number states (MAP) curated for CNV segments on BTA29 related to BFT using mixture model assignment. (**C**) The adjusted traits against signals (LogRR) for CNV segments related to MS at 4 Mb on BTA26. (**D**) The adjusted

traits against copy number states (MAP) estimated for CNV segments on BTA26 related to MS using mixture model assignment.

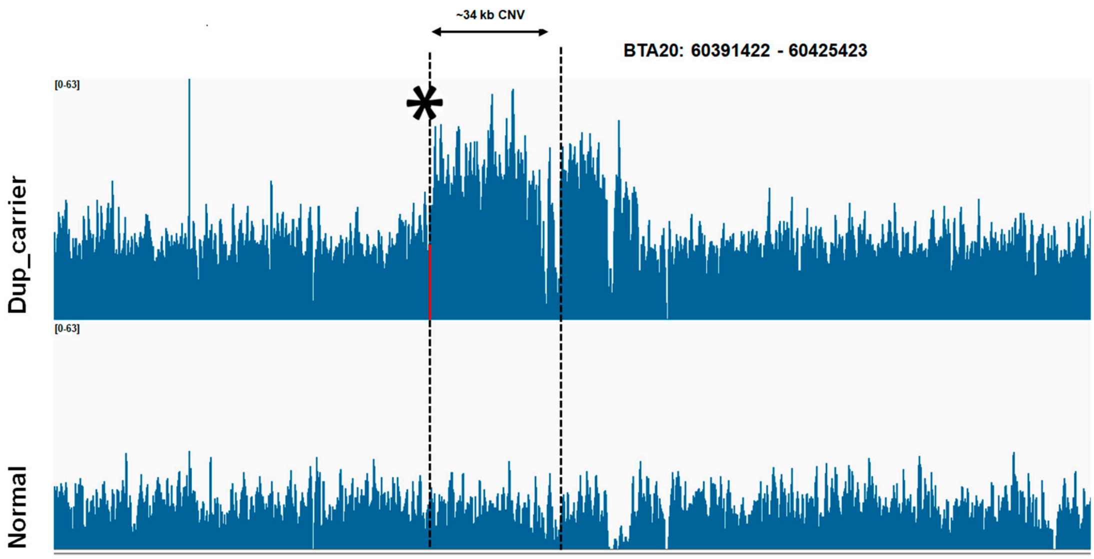

Figure S5. Identification of the 34 kb duplication (BTA20:60391422-60425423) based on WGS of a subject using the IGV program. The lead SNP (red) is marked with a black asterisk.

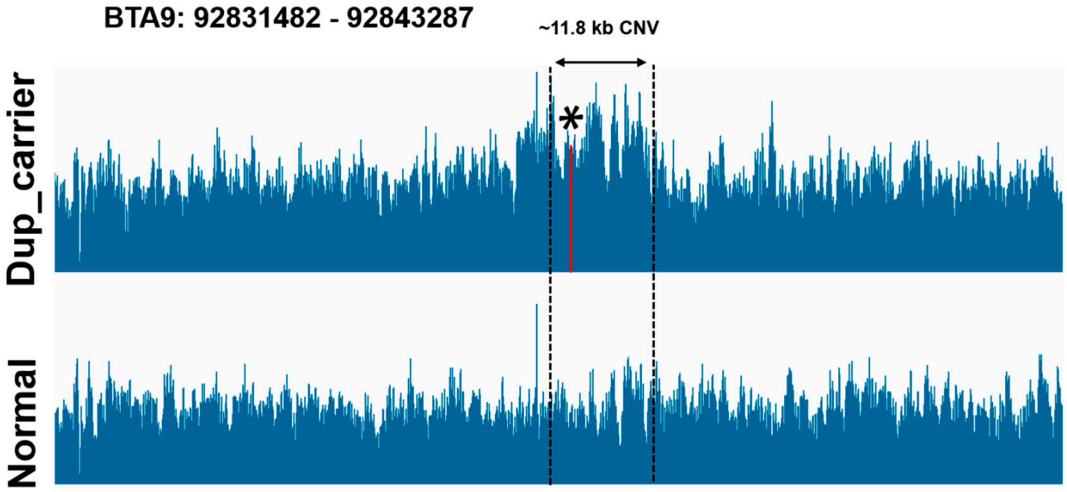

Figure S6. Identification of an 11.8 kb duplication (BTA9:92831482-92843287) based on WGS of a subject using the IGV program. The lead SNP (red) is marked with a black asterisk.

Table S1. Descriptive statistics of six meat quality traits.

Trait	Number	Mean	Min	Max	SD	Heritability
MC	1248	5.896	1	7	1.056	0.132719
FC	1193	1.733	1	5	0.744	0.397855
PH	1211	5.62	4.51	7.16	0.363	0.012799
MS	1253	5.084	1	6	0.97	0.167825
REA	726	75.14	31.84	107.36	11.4805	0.182386
BFT	783	2.519	0.9	5.84	0.881	0.188646

Note: Min: minimum; Max: maximum; SD: standard deviation.

Table S2. The GO and REAC enrichment analyses of gene mapped by CNV-based GWAS.

Term id	Term Name	Adjusted *p* Value	−log10 of Adjusted *p* Value	Effective Domain Size	Intersections
GO:0045095	Keratin filament	0.000181	3.7433576	19665	KRT4, KRT78, KRT79
GO:0005882	Intermediate filament	0.000715	3.145567153	19665	KRT4, KRT78, KRT79
GO:0045111	Intermediate filament cytoskeleton	0.001273	2.895228625	19665	KRT4, KRT78, KRT79
GO:0099513	Polymeric cytoskeletal fiber	0.03544	1.450506358	19665	KRT4, KRT78, KRT79
REAC:R-BTA-6805567	Keratinization	0.000612	3.213463001	4908	KRT4, KRT78, KRT79
REAC:R-BTA-1266738	Developmental biology	0.005957	2.224978325	4908	KRT4, KRT78, KRT79

Note: GO: Gene Ontology; REAC: Reactome.

Table S3. STRING enriched pathways and protein domains for CNV candidate genes related to MC.

Term ID	Term Description	FDR	Matching Proteins ID	Matching Proteins
KW-0403	Intermediate filament	0.00082	ENSBTAP00000000815, ENSBTAP00000016832, ENSBTAP00000016839	KRT78, KRT4, KRT79
KW-0416	Keratin	0.0289	ENSBTAP00000000815, ENSBTAP00000016839	KRT78, KRT79
IPR003054	Keratin, type II	0.0006	ENSBTAP00000000815, ENSBTAP00000016832, ENSBTAP00000016839	KRT78, KRT4, KRT79
IPR032444	Keratin type II head	0.0006		
IPR018039	Intermediate filament protein, conserved site	0.0022	ENSBTAP00000000815, ENSBTAP00000016832, ENSBTAP00000016839	KRT78, KRT4, KRT79
IPR039008	Intermediate filament, rod domain	0.003		

Table S4. Overlapped variants compared with CNVR datasets in cattle.

CNV Segment	BTA	Location (bp)	Length (kb)	Type
CNVR1	5	27,087,501-27,090,000	2.5	Both
CNVR2	29	5,172,501-5,175,000	2.5	Duplication
CNVR2	29	5,181,501-5,184,000	2.5	Both
CNVR2	29	5,213,001-5,215,000	2.0	Both
CNVR2	29	5,239,001-5,242,500	3.5	Duplication
CNVR2	29	5,246,501-5,249,000	2.5	Duplication
CNVR2	29	5,259,001-5,261,000	2.0	Both
CNVR2	29	5,268,501-5,277,500	9.0	Both
CNVR8	20	60,405,501-60,410,000	4.5	Duplication
CNVR11	15	47,407,001-47,441,500	34.5	Both
